# Feasibility and Effectiveness Assessment of SARS-CoV-2 Antigenic Tests in Mass Screening of a Pediatric Population and Correlation with the Kinetics of Viral Loads

**DOI:** 10.3390/v13102071

**Published:** 2021-10-14

**Authors:** Marcello Lanari, Giovanni Battista Biserni, Matteo Pavoni, Eva Caterina Borgatti, Marta Leone, Ilaria Corsini, Tiziana Lazzarotto

**Affiliations:** 1 Pediatric Emergency Unit, Department of Medical and Surgical Sciences, IRCCS Azienda Ospedaliero-Universitaria, Polyclinic of St.Orsola, University of Bologna, 40138 Bologna, Italy; marcello.lanari@unibo.it (M.L.); ilaria.corsini2@unibo.it (I.C.); 2 Specialty School of Pediatrics-Alma Mater Studiorum-University of Bologna, 40138 Bologna, Italy; 3 Microbiology Unit, Department of Specialized, Experimental, and Diagnostic Medicine, IRCCS Azienda Ospedaliero-Universitaria, Polyclinic of St.Orsola, University of Bologna, 40138 Bologna, Italy; matteo.pavoni2@unibo.it (M.P.); evacaterina.borgatti@studio.unibo.it (E.C.B.); marta.leone2@studio.unibo.it (M.L.); tiziana.lazzarotto@unibo.it (T.L.)

**Keywords:** SARS-CoV-2, COVID-19, rapid antigenic test, real time RT-PCR, children, mass screening

## Abstract

The gold standard for diagnosis of SARS-CoV-2 infection has been nucleic acid amplification tests (NAAT). However, rapid antigen detection kits (Ag-RDTs), may offer advantages over NAAT in mass screening, generating results in minutes, both as laboratory-based test or point-of-care (POC) use for clinicians, at a lower cost. We assessed two different POC Ag-RDTs in mass screening versus NAAT for SARS-CoV-2 in a cohort of pediatric patients admitted to the Pediatric Emergency Unit of IRCCS—Polyclinic of Sant’Orsola, Bologna (from November 2020 to April 2021). All patients were screened with nasopharyngeal swabs for the detection of SARS-CoV-2-RNA and for antigen tests. Results were obtained from 1146 patients. The COVID-19 Ag FIA kit showed a baseline sensitivity of 53.8% (CI 35.4–71.4%), baseline specificity 99.7% (CI 98.4–100%) and overall accuracy of 80% (95% CI 0.68–0.91); the AFIAS COVID-19 Ag kit, baseline sensitivity of 86.4% (CI 75.0–93.9%), baseline specificity 98.3% (CI 97.1–99.1%) and overall accuracy of 95.3% (95% CI 0.92–0.99). In both tests, some samples showed very low viral load and negative Ag-RDT. This disagreement may reflect the positive inability of Ag-RDTs of detecting antigen in late phase of infection. Among all cases with positive molecular test and negative antigen test, none showed viral loads > 10^6^ copies/mL. Finally, we found one false Ag-RDTs negative result (low cycle thresholds; 9 × 10^5^ copies/mL). Our results suggest that both Ag-RDTs showed good performances in detection of high viral load samples, making it a feasible and effective tool for mass screening in actively infected children.

## 1. Introduction

Since the spread of SARS-CoV-2, the agent of COVID-19, in early 2020, the gold standard test for diagnosis of the infection has consisted of the detection of viral RNA collected on nasopharyngeal swabs using nucleic acid amplification tests (NAAT), such as reverse transcription real time RT-PCR [1,2]. When employed for mass screening, these techniques harbor some limitations including, but not limited to, the need of expensive equipment, certified laboratories and trained technicians. Two other major concerns regarding the feasibility of molecular testing are widely recognized. The first is the additional workload of healthcare workers and technicians; the second is the time needed for the results which depends on the availability of the healthcare workers, which tends to fluctuate between 4 and 12 h. In cases whereby workload exceeds resources, such time is needed only for transporting specimens to reference laboratories and its processing. In a hospital setting, this implies a delay for correct allocation of patients in order to minimize nosocomial SARS-CoV-2 transmission, whereas, for out-patient care, this means a considerable postponement of in-person attendance of social communities, including daycares, schools and workplace.

Rapid antigen detection kits (Ag-RDTs), may offer advantages over real time RT-PCR; they generate results in 10 to 30 min, can be used both as laboratory-based test or point-of-care (POC) use for clinicians, and, generally, do so at a lower cost. The European Center for Disease Prevention and Control (ECDC) expects benefits from the implementation of Ag-RDTs [3,4] for the prompt clinical management of cases with COVID-19-compatible symptoms at admission, early detection and isolation of cases and contact tracing.

Although, in line with ECDC and WHO, Italy still considers molecular testing as the gold standard for SARS-CoV-2 detection in nasopharyngeal swabs, it has started taking into consideration Ag-RDTs for screening of symptomatic cases up to five days from symptom onset and with either antigenic or molecular confirmation test, in case of negative result [5,6]. Screening with Ag-RDTs of asymptomatic patients is suggested when the prevalence of infection is suspected to exceed 10% in the population. In Italy, antigenic testing was also suggested as a single assay in mass screening of low-risk adults [7], but still, the rate of confirmatory molecular testing nearly reaches 9%. To date, few national experiences include data on children. The aim of this study is to assess feasibility and effectiveness of SARS-CoV-2 point-of-care antigenic tests as a mass screening tool in a cohort of pediatric patients with a low prevalence of SARS-CoV-2 infection.

## 2. Materials and Methods

### 2.1. Study Design and Participants

The target population comprised all pediatric patients< 18 years old admitted to the Pediatric Emergency Department (PED) of IRCCS—Polyclinic of Sant’Orsola in Bologna, Italy, regardless of the cause of admission, the symptoms in question and contact history. Patients were consecutively enrolled from 1 November 2020 to 9 April 2021. The PED of IRCCS—Polyclinic of Sant’Orsola serves a metropolitan area with a population of over 130,000 children and adolescents; in addition, it represents a regional hub for most of the pediatrics branches. In this period, the population presented with a variety of infections- and noninfections-related complaints, both urgent and postponable. Social distancing and protective measures required during SARS-CoV-2 pandemic made outpatient consultation to family physician or pediatrician less accessible, arising less concerns in caregivers to bring their children to PED. Lockdown decreased environmental exposures and the incidence of seasonal infections [8,9]. Moreover, protracted and forced home-stay may have risen the accesses in the PED for functional affections, especially in adolescents [10].

Finally, our PED was the only institution in charge of collecting nasopharyngeal swabs in infants younger than one year of age. We therefore assume that the population assessed in our PED for the duration of the study may have reflected free-living population more than previously.

### 2.2. Procedures and Measurements

#### Testing for SARS-CoV-2 with Antigenic and Qualitative Real Time RT-PCR

During the COVID-19 pandemic, together with the physical examination, pediatric patients admitted to the PED were routinely screened with nasopharyngeal swabs for the detection of SARS-CoV-2-RNA.

Additionally, a second swab was collected from each patient that was immediately tested in PED with COVID -19 Ag FIA and processed with the STANDARD F200 analyzer (SD BIOSENSOR Inc., Yeongtong-gu, Suwon-si, Gyeonggi-do, Korea) or with AFIAS COVID-19 Ag (Boditech Med Inc., Dongnae-myeon, Chuncheon-si, Gang-won-do, Korea). COVID-19 Ag FIA was employed first; when the supply of these test was over, on 15 December 2020, samples were tested with AFIAS COVID-19 Ag until the end of the study.

Both tests are fluorescent immunoassays for the qualitative detection of specific nucleocapsid protein of SARS-CoV-2 present in human nasopharynx. The tests’ procedures and the interpretation of results adopted were reported in the manufacturer instructions for all the assays. The result of the positivity or negativity was cut-off index (COI) ≥ 1 and COI < 1, respectively. The swab for the molecular detection was sent to the Regional Reference Laboratory for Emerging Microorganism (Unit of Microbiology of IRCCS—Polyclinic of Sant’Orsola in Bologna, IT) according to regional protocol and processed with one of the following qualitative real time RT-PCR:

- DiaSorin Molecular Simplexa™ COVID-19 Direct assay system (Diasorin Cypress, CA, USA), which rapidly amplifies two targets of the SARS-CoV-2 genome (the S gene and the ORF1ab gene) without RNA extraction. The assay also reveals the presence of host mRNA in the same reaction to confirm the correct execution of the test. Viral RNA was detected via Liaison^®^MDX plus Direct amplification disc. The final Limit of Detection (LoD) of the test was 500 copies/mL with a Negative Percent Agreement (NPA) and a Positive Percent Agreement (PPA) of 100% [11,12].
- Xpert^®^ Xpress SARS-CoV-2 (Cepheid, Sunnyvale, CA, USA), which rapidly amplifies two targets of the SARS-CoV-2 genome (the E and N2 genes), without RNA extraction, using a proprietary cartridge technology. The LoD was 0.0050 PFU/mL and 0.0200 PFU/mL for N2 and E, respectively, with a PPA of 97.8% (95%CI: 88.4–99.6%) and an NPA of 95.6% (95% CI: 85.2–98.8%). The assay also reveals the presence of host mRNA in the same reaction to confirm the correct execution of the test.

According to WHO and ECDC, real time RT-PCR is the gold standard test for the detection of SARS-CoV-2-RNA [1,2].

Samples showing a positive result for both viral targets were considered positive. Samples with either a single positive target or with Cycle Threshold (CT) value≥ 35 were considered weak reactive [13].

### 2.3. Quantitative Real Time RT-PCR

SARS-CoV-2 RNA (target genes RdRp and ORF8) was extracted, amplified and quantified on the ELITe InGenius platform (ELITechGroup Molecular Diagnostics, Turin, Italy), a fully automated sample-to-result PCR system. RNA was extracted from a Universal Transport Medium (i.e., UTM, COPAN Italy S.p.A.) using the ELITe InGenius total nucleic acid extraction kit specifications with all parameters preprogrammed. SARS-CoV-2 RNA was detected and quantified with a real time PCR assay (SARS-CoV-2 ELITe MGBKit, ELITechGroup Molecular Diagnostics, Turin, Italy) according to the manufacturer’s package insert.

The viral load was reported as number of copies/mL for all body fluids examined. The Lower Limit of Quantification (LLoQ) was 250 copies/mL in this test; the diagnostic specificity and sensitivity were 100% in association with UTM.

### 2.4. Statistical Analysis

Categorical variables were presented as frequencies or percentages; continuous variables with normal distribution were presented as the mean values, standard deviations, and confidence intervals; continuous variables, not normally distributed, were presented as median and interquartile range. Differences in sample means were compared using an independent sample two-tailed Student’s test or Mann–Whitney test, when appropriate.

Contingency tables were built comparing the performance each Ag-RDT with molecular testing. Sensitivity, specificity, positive and negative predictive value (PPV and NPV) were calculated, together with 95% confidence intervals (CI).

A ROC curve was built in order to investigate cut-off values of COI with different sensitivity and specificity and overall accuracy (for both Ag-RDT tests) in comparison with RT-PCR. Cases missing either COI or any swab result were corrected listwise and were not included in statistical analysis.

A type II error of 0.2, an α value of 0.05 (type I error) and a protocol failure of 5% were considered acceptable.

All analyses were conducted with IBM SPSS Statistics for Windows, 22.0 or following versions (IBM Corp., Armonk, NY, USA).

### 2.5. Ethics

The study protocol was approved by the Ethical Committee of IRCCS—Polyclinic of Sant’Orsola (approval code: ICT COVID PS Pediatrico, date: 24 October 2020, Comitato Etico - Area Vasta Emilia Centro CE-AVEC). Written personal informed consent was obtained by the parents or the caregivers of the children involved.

## 3. Results

A total of 1178 patients were enrolled in the study; 386 of them were tested with COVID-19 Ag FIA and 792 with AFIAS COVID-19 Ag; demographic and clinical characteristics are described in Table 1.

### 3.1. COVID-19 Ag FIA Performance

Of the 386 patients that were tested with COVID-19 Ag FIA, 4 were retrieved due to invalid Ag-RDT results. Additionally, in 1 patient, real time RT-PCR result was not available; in the end, a total of 381 cases were included in the analysis. We found concordance with real time RT-PCR results in 96.6% of cases. Of a total of 13 (3.4%) conflicting cases, the one that showed a positive antigenic test and a negative molecular test presented a COI of 1.26. As previously shown in adults [7], COI ranging from 1 to 3 harbors 65.8% of false positive results. Moreover, the patient suffered from acute tonsillitis and upper respiratory tract infection of presumed viral origin. Even though the agent was not identified, the disagreement could have arisen from antigenic cross-reaction.

Regarding the other 12 that had negative COVID-19 Ag FIA test and a positive or weak reactive molecular result (patients from 1 to 12, see Table 2) 10 had CT ≥ 34 for all target genes and both procedures. In cases with only residual amount of one gene target, the possibility of viral isolation is less than 3% and has been associated with the inability of the host to excrete infective viral particles [13,14]. A total of 8 out of 10 patients (patients from 1 to 5 and from 10 to 12) had either no previous contact with a positive COVID-19 case or a previous positive real time RT-PCR result at least 10 days before antigen testing in PED. Concerning the remaining two cases (patients 8 and 9), both had a CT ≤ 35; one had a contact with a positive family member two days before, and the other had a last contact with a known positive patient three weeks before testing. Neither of these two patients developed COVID-19.

**Figure 1:**
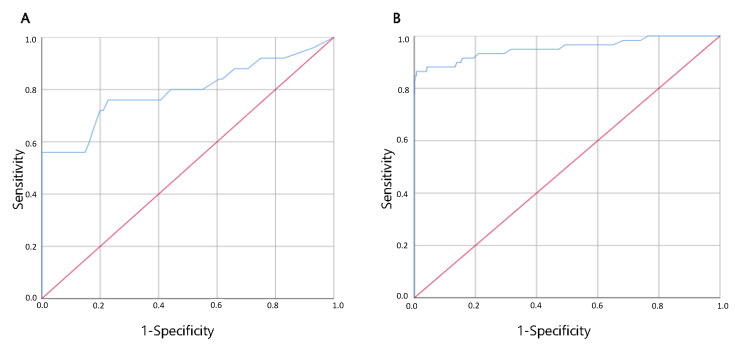
On the left (**A**), ROC curve for COVID-19 Ag FIA Ag-RDT, compared to real time RT-PCR on the right (**B**), ROC curve for AFIAS COVID-19 Ag compared to real time RT-PCR. On the x axis, 1-specificity; on y axis, sensitivity.

The data on COVID-19 Ag FIA tests were reported in the contingency Table 3 (A and B); sensitivity, specificity, PPV and NPV were calculated both for the total of valid assays (Table 3 A and after exclusion of the 10 samples in which SARS-CoV-2-RNA was found in traces (Table 3 B. The overall accuracy of the test is reported in Figure 1A. The area under the ROC curve was shown to approximate 80% (95% CI 0.68–0.91), with a standard error of 0.06.

### 3.2. AFIAS COVID-19 Ag Performance

Analogously, of the 792 patients that were tested with “AFIAS COVID-19 Ag”, 4 were retrieved due to invalid Ag-RDT results, 23 real time RT-PCR results were not available; thus, 765 cases in total were included in the analysis. Concordance was observed with real time RT-PCR results in 97.4% of cases. Conflicting results were 20 (2.6%) in total, of which 12 had positive antigenic test but negative molecular, with a COI ranging from 1.03 to 6.52, again harboring a high rate of false positive results. A total of 8 out of 12 patients had at least an infection-related symptom (fever, pharyngitis or headache) and one that had diarrhea was co-infected with Adenovirus, supporting once more that the disagreement may arise from antigenic cross-reaction.

Eight cases had negative AFIAS COVID-19 Ag test and a positive or weak reactive molecular test (patients from 13 to 20, see Table 2), such as COVID-19 Ag FIA. Three of these eight patients had a previous real time RT-PCR result at least 2 weeks before antigen testing in the PED (patients 13, 14, 15). One case had a CT of 32 without known contacts with COVID-19 positive patients but with symptoms at admission related to urinary tract infections (case 19). During hospital stay, more molecular assays resulted negative and took this into consideration; it was suspected a previous SARS-CoV-2 asymptomatic infection.

The data on AFIAS COVID-19 Ag tests are reported in the contingency Table 4 A, B; then, sensitivity, specificity, PPV and NPV were calculated both for the total of valid assays (Table 4 A) and after exclusion of the seven samples in which SARS-CoV-2-RNA was detected with a CT ≥ 35 for all target genes and both procedures (Table 4 B). The overall accuracy of the test is reported in Figure 1B. The area under the ROC curve was 95.3% (95% CI 0.92–0.99) with standard error of 0.02.

### 3.3. Comparison between Antigenic Test and Quantitative Real Time RT-PCR

Table 2 summarizes the characteristics of the 20 patients with a positive or weak positive qualitative real time RT-PCR result but negative antigenic tests in terms of viral loads and epidemiological link. Kits employed for antigen detection and qualitative PCR with COI and CT, respectively, are also reported in the table. The means of viral loads were comparable (not different by independent sample Student’s test) between patients tested with COVID-19 Ag FIA and AFIAS COVID-19 Ag.

**Figure 2:**
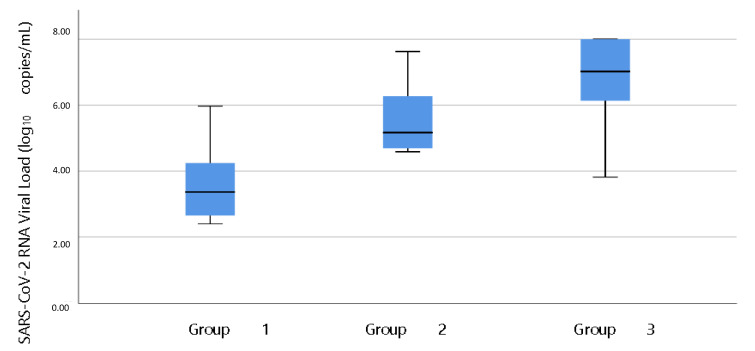
BOX Plot comparing cut-off index (COI) groups with viral loads. Horizontal lines represent mean, blue squares account for the interquartile range and vertical bars represent the lower and upper limits of values (range). Group 1, COI < 1; Group 2, 1 ≤ COI < 12 and Group 3, COI ≥ 12.

Cut-off index obtained with either COVI-19 Ag FIA or AFIAS COVID-19 Ag antigenic test were compared to viral loads obtained with quantitative real time RT-PCR (Figure 2). COI were grouped as follows: group 1, COI < 1; group 2, 1 ≤ COI < 12 and group 3, COI ≥ 12.

## 4. Discussion

The gold standard test for diagnosis of SARS-CoV-2 infection relies on NAAT from the nasopharynges of patients and is mandatory for COVID-19 management. Serological assays have important application for epidemiological studies [15].

A short turnaround time could prevent SARS-CoV-2 diffusion both in healthcare facilities and in the community. In one modeling study, same-day results were predicted to avert 80% of new transmissions, whereas a 7-day delay prevented only 5% [16].

Molecular results obtained via real time RT-PCR demonstrated the detection of viral genome also in individuals that harbor low viral loads, most likely representing the final stages of the infection [17]. Moreover, disagreement on real time RT-PCR results lies on CT values; CT values of 21–18 most likely reflect the 10^5^ to10^6^ RNA copies/mL, quantities below which virus cultures usually become negative and transmission risks are very low [18]. Some laboratories place this threshold at a CT of 30. Currently, there is no international standardization between laboratories and assays, leaving CT calibration with viral load poorly reported and easy to misunderstand [18].

Subgenomic RNA is present only in cells that are infected by the virus and actively transcript the viral genome. A previous study showed that the ratios of mean normalized subgenomic mRNA per genome were about 0.4% [19], starting from day 5 after symptoms onset and declined further the following days. The proportion of genomic RNA remains high in the upper respiratory tract and is detected by PCR, explaining the long wash-out of viral genome. The extent to which the persistency of SARS-CoV-2-RNA in nasopharyngeal swabs may reflect infectivity has been further clarified. Patients who recovered from COVID-19 showed subgenomic RNA in nasopharyngeal swabs up to 79 days from the first positive molecular test, but none were found of the swabs with a CT > 30 [20]. These limits may deeply influence quarantine measures for postinfective COVID-19 patients.

Moreover, the early phase of the infection represents a limitation for real time RT-PCR, showing false negative or positive weak results in actively infected patients [21].

Conversely, though lateral flow antigen testing (LFT) for SARS-CoV-2 has been widely commercialized for its use in airports, pharmacies, schools and universities, it has been questioned for its degree of sensitivity, which has shown not to meet the minimal performance requirements of WHO and ECDC [22,23]. For instance, in the Liverpool pilot, LFT is likely to detect around a third and, at most, half of the PCR positive cases [24].

To summarize, in mass screening, RT-PCR seems to be excessively sensitive, whereas LFT lacks sensitivity. Considering these recent findings, the main question remains open: which test is the best in a mass screening context for prompt detection of actively infected cases?

In this study, we assessed the feasibility and effectiveness of SARS-CoV-2 POC-antigenic tests and real time RT-PCR in a mass screening of pediatric population. Three commercially available real time RT-PCR kits with different primer set sequences were used. Literature data demonstrated that the concurrent use of different platforms does not compromise RNA-SARS-CoV-2 detection. In contrast, the availability of multiple systems enabled us to increase testing capacity and implement testing algorithms [25].

In the early phases of infection, both antigenic tests showed less sensitivity; COVID-19 Ag FIA and AFIAS COVID-19 Ag (patients 8 and 16, Table 2) showed one false negative result not detecting viral proteins. This disagreement has been already observed and may reflect some inability of Ag-RDTs to detect antigen in early phases of infection [5].

Accordingly, positive real time RT-PCR assays with CT values > 30 were shown to be common shortly after symptom onset. Although Ag-RDT showed a lack of sensitivity compared to NAAT to identify individuals with a low viral load, this represents a limit also of the latter method, not to mention that the actual infectivity of such individuals has yet to be proven [26].

CT ≥ 35 is considered a weak positive sample for SARS-CoV-2 [27], because the ability to create cytopathic effect on the medium and isolate the virus in the presence of a CT equal or greater than 35 is less than 3% [13] and, in samples with a viral load < 10^6^ copies/mL, it was not possible to cultivate the virus [17,19].

As reported in Table 2, none of our cases with positive real time RT-PCR and negative Ag-RDT with AFIAS COVID-19 Ag showed >10^6^ copies/mL, and all showed CT > 30, whereas, in the group of COVID-19 Ag FIA, four samples were not available to determine the viral load with quantitative real time PCR. In five out of the other eight cases, viral loads were detected with a number of copies lower than 10^6^, and four of them showed an overall CT > 30. The remaining case presented a CT of 26 for both target genes.

Evidence suggests that, when replication of the virus is controlled by the immune system, the viral loads in nasopharynx of patients drop to levels at which individuals are not likely to infect others [17]. That occurs approximately 10 days from onset of symptoms, but afterwards, real time RT-PCR may still be positive.

Accordingly, for individuals previously infected with the virus, an alternative approach from real time RT-PCR is needed [17]. Detecting SARS-CoV-2 RNA from a previous infection in the nasal cavity may not always relate to current infectivity. Nevertheless, if postinfectious individuals test positive, prolonged quarantine is warranted up to 21 days, meaning a net loss to the health, social and economic well-being of communities at large [22].

Schools and universities can deliver the most basic human right to education and simultaneously keep students and staff safe. Following ECDC recommendations, in many countries, when there is a suspected COVID-19 case that tests positive with NAAT, isolation and testing are recommended for the entire group and those in close contact. Readmission is then possible after 10 days with a negative test (Ag RDT or NAAT) or after 14 days irrespective of testing, whereas the index case requires a negative real time RT-PCR test starting from at least three days after symptoms subsides [28,29]. Furthermore, another important aspect is the use of a test that allows a safe readmission in community of a previously COVID-19 case after a time frame that does not seem to be always correlate with the results of real time RT-PCR. Nevertheless, ECDC considers the need for confirmatory testing when rapid antigen tests are employed in settings of low infection prevalence [30].

ECDC suggests the use of Ag-RDTs tests with a performance closer to real time RT-PCR, which approximates ≥90% sensitivity and ≥97% specificity, while WHO recommends ≥80% sensitivity and ≥97% specificity [4,31]. We obtained an agreement rate over 95% of Ag-RDT to RT-PCR. It could allow a capillary control on the COVID-19 diffusion and a lightening of workload in the laboratories. The short turnaround time allows for results within the same consultation, warranting prompt decisions on quarantine without precautionary isolation for the time before NAAT results are ready. Focusing on our results, we found the required performance (both for ECDC and WHO) with AFIAS COVID-19 Ag, identifying it as a suitable, rapid, affordable, precise and easy-to-use tool for the detection of the infection of SARS-CoV-2 in children for mass screening. Furthermore, in our experience in the setting of a known previous COVID-19, the Ag-RDTs seem to reflect better than PCR the actual infectivity among children.

To our knowledge, this is one of the largest studies comparing Ag-RDTs with NAATs in a pediatric population; however, there are some limitations. In particular:

- In that period of time, the prevalence of the infection fluctuated but remained lower than 10%;
- This fluctuation prevented the examiners from drawing conclusions about statistical and epidemiological data;
- The number of children tested with either Ag-RDT was unbalanced due to the different manufactures’ supply. This circumstance gave more statistic impact to the AFIAS COVID-19 Ag kit;
- Only few positive cases (about 6.8% of the total for COVID-19 Ag FIA and 7.7% for AFIAS COVID-19 Ag) have emerged in our cohort; such a small sample prevented us from drawing conclusions on the actual infectivity in our community.

In conclusion, in this phase of going back to normality from the pandemic, the role of mass screening requires central attention. The best way of keeping educational and social institutions open for in-person learning is to control transmission of SARS-CoV-2 in the wider community and to ensure rapid identification of infected individuals in order to promptly support timely public health responses. A faster, point-of-care, sensitive and more accessible test for the diagnosis, screening and contact tracing of SARS-CoV-2 could have an important impact, especially on education, social and family activity.

## Figures and Tables

**Table 1:** Demographic and clinical characteristics of the population assessed in PED for the period of the study. SD = standard deviation.

Characteristic	COVID-19 Ag FIA	AFIAS COVID-19 Ag
Sex:		
Male	206	450
Female	180	342
Mean age (SD)	4.6 (4.5)	6.8 (7.26)
Infection-related symptoms:	280	494
Fever	198	345
Pharyngitis	114	83
Cough	94	112
Nasal discharge	87	111
Diarrhea	47	74
Headache	21	54
Anosmia/Ageusia	2	5
Asthenia	14	19
Noninfection-related symptoms:	106	298
Results:		
Positive	15	63
Negative	367	725
Invalid	4	4

**Table 2:** Viral loads and epidemiological link of patients with positive qualitative real time RT-PCR and negative Ag-RDTs. Detection of SARS-CoV-2 RNA by quantitative real time RT-PCR analyzing the same specimens used for qualitative real time RT-PCR. The table shows that the patients in which the Ag-RDTs failed to detect the virus have either a previous positive molecular result dating at least 10 days before or no contact history with an index case or are previous COVID-19 patients or have a recent onset of COVID-19-compatible symptoms. NA = not available, ND = not detected.

Patient	Ag-RDT Kit		Qualitative Real Time RT-PCR				Quantitative Real Time RT-PCR	Epidemiological Link
		COI	CT-Value				Copies/mL	
			S *	ORF1ab *	E ^°^	N2 ^°^	RdRp&ORF8 ^§^	
1	COVID-19 Ag FIA	0.24	34	35	-	-	NA	11 days from previous positive result
2	COVID-19 Ag FIA	0.3	-	-	34	35	NA	10 days from previous positive result
3	COVID-19 Ag FIA	0	-	-	38	39	ND	11 days from previous positive result
4	COVID-19 Ag FIA	0.07	-	-	35	0	2.3 × 10^3^	13 days from previous positive result
5	COVID-19 Ag FIA	0.24	38	0	-	-	ND	23 days from previous positive result
6	COVID-19 Ag FIA	0.03	37	39	-	-	1 × 10^3^	8 days from onset of symptoms
7	COVID-19 Ag FIA	0.05	35	0	-	-	NA	0 days from onset of symptoms
8	COVID-19 Ag FIA	0.25	32	35	-	-	2.3 × 10^4^	2 days from last contact with a confirmed COVID-19
9	COVID-19 Ag FIA	0.01	-	-	26	26	9 × 10^5^	21 days from last contact with a confirmed COVID-19
10	COVID-19 Ag FIA	0.22	38	0	-	-	4.5 × 10^2^	no contact history nor COVID-19 symptoms
11	COVID-19 Ag FIA	0.12	38	0	-	-	ND	no contact history nor COVID-19 symptoms
12	COVID-19 Ag FIA	0.26	-	-	35	0	NA	no contact history nor COVID-19 symptoms
13	AFIAS COVID-19 Ag	0.49	-	-	35	36	NA	25 days from previous positive result
14	AFIAS COVID-19 Ag	0.55	-	-	35	0	detected < 250	15 days from onset of symptoms
15	AFIAS COVID-19 Ag	0.41	-	-	37	0	ND	22 days from onset of symptoms
16	AFIAS COVID-19 Ag	0.35	-	-	37	0	1.7 × 10^4^	0 days from onset of symptoms
17	AFIAS COVID-19 Ag	0.32	38	0			NA	3 days from onset of symptoms
18	AFIAS COVID-19 Ag	0.63	35	35			1.1 × 10^4^	no contact history nor COVID-19 symptoms
19	AFIAS COVID-19 Ag	0.86	-	-	0	32	NA	no contact history nor COVID-19 symptoms
20	AFIAS COVID-19 Ag	0.61	0	35			NA	no contact history nor COVID-19 symptoms

* DiaSorin Molecular Simplexa™ COVID-19: S and ORF1ab genes targets; ° Xpert^®^ Xpress SARS-CoV-2: E and N2 genes targets; **^§^** SARS-CoV-2 ELITe MGB Kit: RdRp and ORF8 genes targets. COI: cut-off index.

**Table 3:** Cross-table, sensitivity, specificity, positive predictive value (PPV), negative predictive value (NPV) for COVID-19 Ag FIA Ag-RDT.

A					B				
		Real Time RT-PCR					Real Time RT-PCR		
		Positive	Negative	tot			Positive	Negative	tot
Ag-RDT	Positive	14	1	15	Ag-RDT	Positive	14	1	15
	Negative	12	354	366		Negative	2	354	356
	tot	26	355	381		tot	16	355	371
Sensitivity = 53.8% (CI 35.4–71.4%), Specificity = 99.7% (CI 98.4–100%), PPV= 93.3% (CI 65.7–99%), NPV=96.7% (CI 96.7–98.2%).					Sensitivity = 87.5% (CI 61.6–98.4%), Specificity = 99.7% (CI 98.4–100%), PPV = 93.3% (CI 66.2–98%), NPV = 99.4% (CI 98–99.8%).				

**Table 4:** Cross-table, sensitivity, specificity, positive predictive value (PPV), negative predictive value (NPV) for AFIAS COVID-19 Ag-RDT.

A					B				
		Real Time RT-PCR					Real Time RT-PCR		
		Positive	Negative	tot			Positive	Negative	tot
Ag-RDT	Positive	51	12	63	Ag-RDT	Positive	51	12	63
	Negative	8	694	702		Negative	1	694	695
	tot	59	706	765		tot	52	706	758
Sensitivity = 86.4% (CI 75–93.9%), Specificity = 98.3% (CI 97.1–99.1%), PPV = 81% (CI 70.6–88.3%), NPV = 98.9% (CI 97.9–99.4%)					Sensitivity = 98.1% (CI 89.7–99.9%), Specificity = 98.3% (CI 97.1–99.1%), PPV = 81% (CI 70.8–88.2%), NPV = 99.9% (CI 99–100%)				

## Data Availability

The data presented in this study are available on request from the corresponding author. The data are not publicly available due to Ethics Committee restrictions. Written consent from the Promoter of the study is required for data access.
